# Alzheimer Disease Biomarkers and Subjective Cognitive Decline Among Hispanic and/or Latino Adults

**DOI:** 10.1001/jamanetworkopen.2025.31038

**Published:** 2025-09-05

**Authors:** Freddie Márquez, Wassim Tarraf, Kevin Gonzalez, Deisha F. Valencia, Ariana M. Stickel, Natasha Z. Anita, Daniela Sotres-Alvarez, Bonnie E. Levin, Michael A. Yassa, Haibo Zhou, Martha Daviglus, Amber Pirzada, Zachary T. Goodman, Bharat Thyagarajan, Linda C. Gallo, Hector M. González

**Affiliations:** 1 Department of Neurosciences, University of California, San Diego, La Jolla; 2 Institute of Gerontology and Department of Healthcare Sciences, Wayne State University, Detroit, Michigan; 3 Department of Psychology, San Diego State University, San Diego, California; 4 Department of Biostatistics, University of North Carolina at Chapel Hill; 5 Department of Neurology, University of Miami, Miami, Florida; 6 Department of Neurobiology and Behavior and Center for the Neurobiology of Learning and Memory, University of California, Irvine; 7 Institute for Minority Health Research, College of Medicine, University of Illinois at Chicago; 8 Department of Psychology, University of Miami, Miami, Florida; 9 Department of Laboratory Medicine and Pathology, University of Minnesota, Minneapolis; 10 Shiley-Marcos Alzheimer’s Disease Research Center, University of California, San Diego, La Jolla

## Abstract

**Importance:**

Subjective cognitive decline (SCD) may be an early indicator of Alzheimer disease and related dementias (ADRD), yet its association with plasma biomarkers remains unclear among middle-aged and older adults (aged 50-86 years).

**Objective:**

To examine associations between plasma biomarkers of amyloid, tau, neuroaxonal damage, and glial activation with SCD in a heterogeneous cohort of Hispanic and/or Latino adults.

**Design, Setting, and Participants:**

This cross-sectional study used survey-weighted data from the Study of Latinos–Investigation of Neurocognitive Aging, an ancillary study of the Hispanic Community Health Study/Study of Latinos. Participants were aged 50 to 86 years and resided in 4 major US cities. Data were collected from 2016 to 2018 and analyzed between December 2024 and June 2025.

**Exposure:**

Plasma biomarkers included amyloid-beta (Aβ_42/40_), phosphorylated tau-181 (ptau-181), neurofilament light chain (NfL), and glial fibrillary acidic protein (GFAP), quantified using Simoa (Quanterix HD-X) and log-transformed (ln) to reduce skewness.

**Main Outcomes and Measures:**

SCD was assessed using the short-form Everyday Cognition Scale (ECog-12), evaluating global-, executive-, and memory-related SCD, and a single-item cognitive concerns question. Survey-weighted linear and logistic regression models tested associations between biomarkers and SCD, adjusting for demographic, cardiovascular, kidney, and *APOE* genotype covariates.

**Results:**

Among 5712 adults (mean [SD] age, 63.47 (8.15) years; unweighted 3663 [53.92%] female), higher ln(ptau-181) was associated with ECog-12 memory (unstandardized β = 0.088; 95% CI, 0.005-0.170). Higher ln(NfL) levels were associated with greater ECog-12 global (unstandardized β = 0.169; 95% CI, 0.074-0.265), executive (unstandardized β = 0.182; 95% CI, 0.087-0.277), and memory (unstandardized β = 0.156; 95% CI, 0.065-0.248) domains. Higher ln(GFAP) levels were associated with greater ECog-12 global (unstandardized β = 0.109; 95% CI, 0.019-0.198) and executive (unstandardized β = 0.121; 95% CI, 0.031-0.211) domains. Ln(Aβ_42/40_) was not associated with SCD domains. Cognitive concerns significantly modified the associations between ln(NfL) and ECog-12 domains, with more pronounced associations among those reporting cognitive concerns. No biomarkers were associated with the single-item cognitive concerns score.

**Conclusions and Relevance:**

In this study of middle-aged and older Hispanic and/or Latino adults, plasma biomarkers of p-tau181, NfL, and GFAP, but not Aβ_42/40_, were associated with greater SCD. These findings underscore their potential utility in early ADRD detection strategies.

## Key Points

**Question:**

Are plasma biomarkers for Alzheimer disease associated with subjective cognitive decline (SCD) in Hispanic and/or Latino adults?

**Findings:**

In this cross-sectional study of 5712 Hispanic and/or Latino adults aged 50 to 86 years, higher phosphorylated tau-181, neurofilament light chain, and glial fibrillary acidic protein levels were associated with greater SCD, assessed with the Everyday Cognition Scale, but not cognitive concerns. No associations were found for amyloid-beta levels.

**Meaning:**

These findings suggest that tau pathology, neurodegeneration, and neuroinflammation may reflect early cognitive changes and help identify at-risk individuals among Hispanic and/or Latino adults.

## Teaser

This cross-sectional study evaluates associations between plasma biomarkers of amyloid, tau, neuroaxonal damage, and glial activation with subjective cognitive decline in a heterogeneous cohort of Hispanic and/or Latino adults.

## Introduction

As the population ages, affordable, population-level risk-markers are needed to screen for early and presymptomatic stages of Alzheimer disease (AD) and related dementias (ADRD). Self-reports of cognitive concerns experienced by aging adults are increasingly recognized as potentially important indicators of cognitive symptoms for clinicians. Subjective cognitive decline (SCD), a condition first described by researchers in 2014, refers to the perceived decline in cognitive function in comparison with a previously normal status by the participant or an informant.^1^ It is increasingly recognized as an early marker of cognitive decline, preceding mild cognitive impairment (MCI) and ADRD.^2,3^ The Subjective Cognitive Decline Initiative framework and characterization for SCD describes it as a broad condition with varied trajectories and underlying causes.^1,4^

Cost-effective ADRD markers are especially needed for historically underrepresented groups, including Hispanic and/or Latino adults, which are the second largest racial or ethnic population (19.5%) in the US and are projected to have the largest increase in ADRD prevalence in the coming decades.^5,6^ This population also has a high prevalence of cardiometabolic comorbidities. Our prior work^7^ showed that SCD is associated with objective cognitive decline over 7 years in community-dwelling middle-aged and older Hispanic and/or Latino adults, supporting its potential as an important assessment in this population.

Integrating blood-based biomarkers with SCD assessments in primary care could enhance early identification and risk stratification of ADRDs. These biomarkers are becoming increasingly accessible and affordable, improving reach to medically underserved populations. Updated AD diagnostic criteria identify core biomarkers of AD neuropathology, including amyloid-beta (A category; Aβ_42/40_) and phosphorylated tau variants (T category; ptau-181).^8^ Additionally, biomarkers of neurodegeneration or neuronal injury (N category; eg, neurofilament light chain [NfL]) and inflammation (I category; eg, glial fibrillary acidic protein [GFAP]) reflect nonspecific processes involved in AD pathophysiology.

The relationship between plasma biomarkers and SCD remains poorly understood. However, there is a need for simple, accurate methods for identifying different underlying neurodegenerative diseases in patients with cognitive symptoms.^9^ Understanding these associations could improve diagnostics, treatment approaches, and interventions,^10^ but requires investigations in large, heterogeneous populations before implementation into clinical practice. Since AD is a progressive disease that develops over decades,^11^ middle-age may be a key window for lifestyle and medical interventions to reduce ADRD risk.

In this study, we examined cross-sectional associations between plasma biomarkers and SCD in middle-aged and older Hispanic and/or Latino individuals. Although SCD is typically defined in cognitively unimpaired individuals, we included participants with and without impairment to reflect the continuum of clinical practice presentation. We also investigated potential effect modification by age, sex, and cognitive status. We hypothesized that higher ptau-181, NfL, and GFAP levels and lower Aβ_42/40_ levels would be associated with greater global and domain-specific (executive function and memory) SCD.

## Methods

### Population

The Hispanic Community Health Study/Study of Latinos (HCHS/SOL) is a probability-sampled, prospective cohort study of 16 415 self-identified Hispanic and/or Latino adults (aged 18-74 years; visit 1 occurred 2008-2011). The HCHS/SOL study uses a complex survey design incorporating stratification, clustering, and sampling weights^12^ to produce representative data for Hispanic and/or Latino adults in 4 targeted US metropolitan areas: Bronx, New York; Chicago, Illinois; Miami, Florida; and San Diego, California. The Study of Latinos–Investigation of Neurocognitive Aging (SOL-INCA; 2016-2018) is an ancillary study of HCHS/SOL, conducted during the second visit to assess neurocognition in a subset of participants who underwent neurocognitive testing at visit 1, completed HCHS/SOL visit 2, and were aged 50 years and older at visit 2. Of the 6377 SOL-INCA participants aged 50 years and older, 151 participants were excluded due to unavailable or unprocessed samples, and 514 participants had missing covariates resulting in a final unweighted analytical sample of 5712 (eFigure 1 in Supplement 1). All participants provided written informed consent, and the study protocol was approved by institutional review boards at all participating institutions. This report follows the Strengthening the Reporting of Observational Studies in Epidemiology (STROBE) guideline for cross-sectional studies.

### Exposures and Outcome Variables

Plasma biomarkers were the primary exposures in this study. Fasting blood samples (approximately 80 mL) were collected using a standardized venipuncture protocol during the HCHS/SOL clinic visit.^13,14^ Biospecimens were processed at each field center into 500 μl serum and plasma aliquots and frozen at −80 °C. Ethylenediaminetetraacetic acid plasma tubes were processed within 15 minutes centrifuged at 3000 × g for 30 minutes at 15 °C.

Frozen plasma samples were shipped on dry ice from the field centers to the University of Minnesota’s Advanced Research and Diagnostic Laboratory. All plasma biomarker concentrations were measured in picograms per milliliter. Aβ_40_, Aβ_42_, NfL, and GFAP were assayed with the Simoa Neurology 4-Plex E Advantage (Quanterix). Simoa ptau-181 Advantage v2 (Quanterix) was used for ptau-181, and a subset of samples were assayed with the NF-light Advantage (Quanterix) for NfL. Aβ_40_ and Aβ_42_ were used to calculate the Aβ_42/40_ ratio. A subsample was retested for reliability and reproducibility (coefficients of variation of the blinded duplicates were <11.3%). All assays were performed on the Simoa platform HD-X platform (Quanterix).^15^

Subjective cognitive decline outcomes were measured at the SOL-INCA visit using the 12-item Everyday Cognition Scale (ECog-12) and cognitive concerns. The ECog-12 assesses cognitively mediated functional abilities in older adults^16,17^ and is widely used as a measure of SCD.^18,19,20,21^ The self-report version of the ECog-12 predicts progression to MCI^22^ and discriminates between dementia and normal cognition.^17^ The ECog-12 was administered in either English or Spanish based on the participants’ language preference. The Spanish version was translated by a certified translator (American Translators Association certified by the State of Florida; Certificate No. 34371), and cultural appropriate recommendations from bilingual and bicultural staff. Participants rated their current ability to perform daily tasks (memory, language, visuospatial, and executive functions) compared with 10 years ago on a scale of 1 to 4 (1, better or no change to 4, consistently much worse). Global, executive, and memory SCD scores (ie, ECog-12 global, executive, and memory) were calculated by averaging item scores and *z* scored ([Χ_i_-Mean]/SD) using the SOL-INCA target population means and SDs. There is evidence that an individual’s concerns (or worries) predict the likelihood of cognitive decline or conversion to dementia.^23^ Thus, cognitive concern (worry) is included as a feature that increases the risk of cognitive decline in a concept called SCD plus.^1,4^ Cognitive concerns were measured based on responding “yes” to the question: “Are you worried or believe that you are having problems with your attention, concentration, or memory?”

### Covariables

Covariables were measured at HCHS/SOL visit 2 (2014-2017), and included age (in years), sex (female and male), level of education (less than high school, high school or equivalent, or more than high school), Hispanic and/or Latino background (Central American, Cuban, Dominican, Mexican, Puerto Rican, South American, or more than 1/other), and field center (Bronx, Chicago, Miami, or San Diego).

Given the literature linking body mass index (BMI; alculated as weight in kilograms divided by height in meters squared), cardiovascular disease (CVD) risk factors, and kidney dysfunction to altered levels of plasma biomarkers and cognitive performance,^24,25,26,27,28^ we added those measures as follows: BMI was defined as underweight (<18.5), normal (18.5-24.9), overweight (25.0-29.9), and obese (≥30.0). Diabetes status was defined per American Diabetes Association criteria (normal glucose regulation, impaired glucose tolerance, or diabetes),^29^ hypertension status per National Health and Nutrition Examination Survey criteria (systolic blood pressure >140 mm Hg and diastolic blood pressure >90 mm Hg or self-reported current antihypertensive medication use),^30^ dyslipidemia status (LDL-cholesterol ≥160 mg/dL [to convert to mmol/L, multiply by 0.0259], or HDL-cholesterol <40 [to convert to mmol/L, multiply by 0.0259], or Triglycerides ≥200 mg/dL [to convert to mmol/L, multiply by 0.0113]) and chronic kidney disease (CKD) status based on estimated glomerular filtration rate (eGFR; CKD epidemiology collaboration creatinine cystatin C formula)^31^ and urine albumin-creatinine ratio (uACR), defined as meeting either condition of eGFR less than 60 mL/min/1.73m^2^ or uACR 30 mg/g or greater. *APOE* genotype was also included, given its association with memory concerns^32,33,34^ and amyloid burden in *APOE* ε4 carriers.^35,36^

### Statistical Analysis

We characterized the SOL-INCA population by outcomes and covariates using survey-adjusted χ^2^ tests and *F* tests to assess differences by cognitive concerns. Survey-weighting was applied in all analyses to reflect the complex design of the sample and account for the probability weights. Primary analyses used natural log-transformed (ln) plasma biomarker concentrations to address skewedness. In supplemental analyses, we also evaluated associations using standardized (*z* scored) biomarker values without log transformation to facilitate interpretability and assess consistency of findings (eTable 2 in Supplement 1). Linear regression examined cross-sectional associations between the plasma biomarkers and ECog-12 outcomes (global, executive, and memory), reporting estimates (unstandardized β) and 95% CIs for 5 models: (1) unadjusted, (2) adjusted for demographics (age, sex, education, Hispanic and/or Latino background, and field center), (3) further adjusted for cardiometabolic factors (BMI, diabetes, hypertension, and dyslipidemia), (4) further adjusted for CKD, and (5) additionally adjusted for *APOE* genotype. We estimated and plotted marginal means and their 95% CIs post hoc (eFigure 2 in Supplement 1). Survey-weighted logistic regression models examined associations between plasma biomarkers and cognitive concerns, reporting odds ratios (OR) and 95% CIs.

We assessed effect modification by age (<65 years or ≥65 years), sex, and cognitive concerns in the association between the plasma biomarkers and ECog-12 (eTable 4 in Supplement 1) using *F* tests for interaction significance. Significant interactions were followed by stratified models and plotted group-specific marginal mean estimates with 95% CIs (eTable 5 and eFigure 2 in Supplement 1).

To ensure that our results were not driven by cognitively impaired individuals and minimize potential sources of bias, we conducted a sensitivity analysis restricted to the cognitively unimpaired subpopulation. MCI was defined using National Institutes of Aging-Alzheimer’s Association criteria.^37^ Fully adjusted model estimates are presented in eTable 6 in Supplement 1. To further assess robustness to model assumptions, we re-estimated associations using survey-weighted generalized linear models (log link and gamma family) to account for the positive skew in the ECog-12 outcomes (eTable 7 in Supplement 1).

A post hoc analysis tested whether the observed associations remained significant after adjusting for self-reported depression (Center of Epidemiologic Studies Depression Scale-10) and anxiety state (State Trait Anxiety Inventory) in a survey linear regression model (eTable 8 in Supplement 1). All analyses accounted for the complex design (probability weights, stratification, and clustering) and were conducted in Stata version 18 (StataCorp) using the survey functionalities for generalizability to the SOL-INCA target population. *P *values for the regression coefficients were obtained from 2-tailed *t* tests, and *P *values for testing the interaction terms from Wald *F* tests. The threshold was set at a statistical significance level of .05. Data were collected from 2016 to 2018 and analyzed between December 2024 and June 2025.

## Results

### Sample Characteristics

Among 5712 Hispanic and/or Latino adults (mean [SD] age, 63.47 [8.15] years; unweighted 3663 [53.92%] female), participants with cognitive concerns differed significantly in age, sex, Hispanic and/or Latino background, education, diabetes status, and cognitive status, but not in field center, BMI, hypertension, dyslipidemia, or CKD (Table 1). We report the means, SDs, and quartiles for all plasma biomarkers and ECog-12 measures (eTable 1 in Supplement 1).

**Table 1.** Descriptive Statistics of SOL-INCA Participants Shown by Self-Reporting Cognitive Concern Groups, SOL-INCA (Unweighted N = 5712)^a^

Characteristic	Participants, % (SE)			*P* value^b^
	No cognitive concerns (n = 1875)	Cognitive concerns (n = 3837)	Total (N = 5712)	
Age, mean (SD), y	62.70 (7.68)	63.88 (8.37)	63.47 (8.15)	<.001
Sex				
Female	46.44 (1.59)	57.75 (1.09)	53.92 (0.88)	<.001
Male	53.56 (1.59)	42.25 (1.09)	46.08 (0.88)	
Hispanic or Latino background				
Dominican	10.96 (1.09)	8.46 (0.83)	9.31 (0.77)	.01
Central American	7.08 (0.83)	7.39 (0.71)	7.29 (0.60)	
Cuban	25.17 (2.31)	27.38 (2.11)	26.63 (1.98)	
Mexican	32.47 (2.10)	33.09 (1.85)	32.88 (1.72)	
Puerto Rican	13.83 (1.04)	15.63 (0.99)	15.02 (0.84)	
South American	5.16 (0.58)	5.23 (0.48)	5.21 (0.41)	
Other or >1	5.33 (0.99)	2.81 (0.46)	3.67 (0.46)	
Education				
Less than high school	33.16 (1.66)	40.88 (1.29)	38.26 (1.09)	<.001
High school or equivalent	19.07 (1.32)	21.11 (0.95)	20.42 (0.77)	
More than high school	47.77 (1.58)	38.01 (1.24)	41.32 (1.04)	
Field center				
Bronx	28.27 (1.97)	25.43 (1.64)	26.39 (1.52)	.26
Chicago	11.12 (1.10)	12.95 (0.94)	12.33 (0.87)	
Miami	36.51 (2.69)	37.39 (2.50)	37.09 (2.37)	
San Diego	24.10 (1.98)	24.23 (1.81)	24.19 (1.69)	
BMI^c^				
<18.5	0.56 (0.19)	0.46 (0.15)	0.50 (0.12)	.94
18.5-24.9	16.57 (1.17)	16.78 (0.90)	16.71 (0.71)	
25.0-29.9	41.06 (1.64)	40.17 (1.21)	40.47 (1.02)	
≥30.0	41.81 (1.62)	42.59 (1.17)	42.32 (1.00)	
Diabetes				
No diabetes	17.00 (1.34)	14.85 (0.73)	15.58 (0.69)	.01
Prediabetes	44.66 (1.63)	41.14 (1.18)	42.33 (0.97)	
Diabetes	38.34 (1.62)	44.01 (1.12)	42.09 (0.96)	
Hypertension	55.99 (1.58)	59.55 (1.14)	58.34 (0.89)	.08
Dyslipidemia	36.96 (1.59)	36.55 (1.19)	36.69 (0.97)	.83
CKD	15.92 (1.24)	18.19 (1.03)	17.42 (0.82)	.16
*APOE* genotype				
ε2/ε2	0.28 (0.14)	0.29 (0.09)	0.28 (0.07)	.09
ε2/ε3	8.10 (0.78)	6.85 (0.57)	7.27 (0.45)	
ε2/ε4	1.18 (0.31)	1.06 (0.20)	1.10 (0.17)	
ε3/ε3	70.77 (1.38)	70.64 (1.11)	70.68 (0.85)	
ε3/ε4	18.96 (1.18)	19.28 (0.92)	19.17 (0.73)	
ε4/ε4	0.71 (0.21)	1.89 (0.32)	1.49 (0.22)	
Cognitive status				
Cognitively unimpaired	95.81 (0.63)	84.61 (0.83)	88.40 (0.59)	<.001

Abbreviations: *APOE*, Apolipoprotein E; BMI, body mass index; CKD, chronic kidney disease; ε, allele; SOL-INCA, The Study of Latinos–Investigation of Neurocognitive Aging.

^a^ Sample size is unweighted; all other reported values are weighted.

^b^ Results are derived from χ^2^ tests and *F* tests using data from SOL-INCA.

^c^ BMI is calculated as weight in kilograms divided by height in meters squared.

### ECog-12 Score Distributions

The ECog-12 global score was highly correlated with both the executive (*r* = 0.94) and memory (*r* = 0.79) subdomain scores. The executive and memory scores were moderately correlated with each other (*r* = 0.65). These strong correlations are expected, given that the global score reflects the mean across all ECog-12 items, including both domains.

### Associations Between Plasma Biomarkers and ECog-12 Scores

In fully adjusted models (Table 2), higher ln(ptau-181) was associated with higher memory ECog-12 scores (unstandardized β = 0.088; 95% CI, 0.005-0.170; *P* = .04). Higher ln(NfL) was associated with higher ECog-12 global (unstandardized β = 0.169; 95% CI, 0.074-0.265; *P* = .001), executive (unstandardized β = 0.182; 95% CI, 0.087-0.277; *P* < .001), and memory (unstandardized β = 0.156; 95% CI, 0.065-0.248; *P* < .001) scores. Higher ln(GFAP) was also associated with higher ECog-12 global (unstandardized β = 0.109; 95% CI, 0.019-0.198; *P* = .02) and executive (unstandardized β = 0.121; 95% CI, 0.031-0.211; *P* = .01) scores. No significant main effects were found between Aβ_42/40_ and ECog-12 outcomes. Associations using standardized, untransformed plasma biomarkers were consistent with the main results (eTable 2 in Supplement 1).

**Table 2.** Associations Between Log-Transformed Plasma Biomarkers and Subjective Cognitive Decline (ECog-12) in the SOL-INCA Population (Unweighted N = 5712)^a^

Biomarker	Model 1^b^		Model 2^b^		Model 3^b^		Model 4^b^		Model 5^b^	
	β (95% CI)	*P* value	β (95% CI)	*P* value	β (95% CI)	*P* value	β (95% CI)	*P* value	β (95% CI)	*P* value
ECog-12 global										
ln(Aβ_42/40_)	−0.139 (−0.306 to 0.027)	.10	−0.087 (−0.251 to 0.078)	.30	−0.065 (−0.230 to 0.100)	.43	−0.052 (−0.218 to 0.114)	.53	−0.043 (−0.209 to 0.122)	.61
ln(ptau-181)	0.130 (0.053 to 0.207)	.001	0.091 (0.012 to 0.171)	.03	0.078 (−0.001 to 0.158)	.05	0.075 (−0.007 to 0.157)	.07	0.066 (−0.016 to 0.148)	.12
ln(NfL)	0.233 (0.151 to 0.314)	<.001	0.176 (0.087 to 0.264)	<.001	0.166 (0.077 to 0.254)	<.001	0.171 (0.075 to 0.267)	<.001	0.169 (0.074 to 0.265)	.001
ln(GFAP)	0.201 (0.119 to 0.282)	<.001	0.109 (0.019 to 0.198)	.02	0.124 (0.035 to 0.212)	.01	0.119 (0.030 to 0.207)	.01	0.109 (0.019 to 0.198)	.02
ECog-12 executive										
ln(Aβ_42/40_)	−0.149 (−0.306 to 0.007)	.06	−0.094 (−0.247 to 0.059)	.23	−0.074 (−0.226 to 0.079)	.34	−0.066 (−0.218 to 0.085)	.39	−0.052 (−0.204 to 0.099)	.49
ln(ptau-181)	0.139 (0.056 to 0.222)	.001	0.106 (0.023 to 0.189)	.01	0.094 (0.010 to 0.177)	.03	0.081 (−0.005 to 0.167)	.07	0.075 (−0.012 to 0.162)	.09
ln(NfL)	0.230 (0.146 to 0.314)	<.001	0.192 (0.103 to 0.281)	<.001	0.187 (0.098 to 0.275)	<.001	0.183 (0.088 to 0.278)	<.001	0.182 (0.087 to 0.277)	<.001
lnvGFAP)	0.179 (0.094 to 0.263)	<.001	0.118 (0.027 to 0.209)	.01	0.137 (0.047 to 0.226)	.003	0.126 (0.037 to 0.215)	.01	0.121 (0.031 to 0.211)	.01
ECog-12 memory										
ln(Aβ_42/40_)	−0.135 (−0.291 to 0.020)	.09	−0.108 (−0.261 to 0.045)	.17	−0.099 (−0.250 to 0.053)	.201	−0.098 (−0.249 to 0.053)	.20	−0.085 (−0.236 to 0.066)	.27
ln(ptau-181)	0.118 (0.044 to 0.193)	.002	0.092 (0.011 to 0.173)	.03	0.086 (0.005 to 0.168)	.04	0.097 (0.014 to 0.179)	.02	0.088 (0.005 to 0.170)	.04
ln(NfL)	0.190 (0.114 to 0.267)	<.001	0.151 (0.064 to 0.238)	.001	0.143 (0.056 to 0.230)	.001	0.159 (0.066 to 0.252)	.001	0.156 (0.065 to 0.248)	.001
ln(GFAP)	0.123 (0.044 to 0.202)	.002	0.045 (−0.043 to 0.132)	.32	0.049 (−0.040 to 0.137)	.28	0.049 (−0.040 to 0.138)	.28	0.037 (−0.052 to 0.127)	.41

Abbreviations: Aβ, amyloid beta; ECog-12, 12-Item form of the Everyday Cognition Scale; executive, executive function; GFAP, glial fibrillary acidic protein; global, global cognition; ln, log transformed; NfL, neurofilament light chain; ptau, phosphorylated tau; SOL-INCA, The Study of Latinos–Investigation of Neurocognitive Aging.

^a^ Results are derived from survey-weighted linear regression estimates (unstandardized β), 95% CIs, and *P* values for associations between natural log-transformed plasma biomarkers and ECog-12 domain scores (global, executive, and memory) across 5 nested models using data from SOL-INCA.

^b^ Model 1 is an unadjusted model; model 2 is adjusted for age, sex, education, Hispanic or Latino background, and field center; model 3 is additionally adjusted for body mass index, diabetes, hypertension, and dyslipidemia; model 4 is additionally adjusted for chronic kidney disease; and model 5 is additionally adjusted for *APOE* genotype.

### Cognitive Concerns Associations

Plasma biomarkers were not associated with self-reported cognitive concerns after full covariate adjustment (Table 3). Results using standardized, untransformed plasma biomarkers were similar (eTable 3 in Supplement 1).

**Table 3.** Associations Between Natural Log-Transformed Plasma Biomarkers and Self-Reported Cognitive Complaints in the SOL-INCA Population (Unweighted N = 5712)^a^

Biomarker	Model 1^b^		Model 2^b^		Model 3^b^		Model 4^b^		Model 5^b^	
	OR (95% CI)	*P* value	OR (95% CI)	*P* value	OR (95% CI)	*P* value	OR (95% CI)	*P* value	OR (95% CI)	*P* value
Cognitive complaints										
ln(Aβ_42/40_)	0.999 (0.744-1.342)	.99	1.098 (0.811-1.488)	.54	1.119 (0.823-1.521)	.47	1.122 (0.821-1.532)	.47	1.153 (0.845-1.575)	.37
ln(ptau-181)	1.222 (1.023-1.461)	.03	1.177 (0.979-1.417)	.08	1.165 (0.968-1.401)	.11	1.164 (0.978-1.387)	.09	1.145 (0.961-1.364)	.13
ln(NfL)	1.216 (1.039-1.422)	.02	1.107 (0.942-1.302)	.22	1.077 (0.910-1.273)	.39	1.077 (0.907-1.279)	.40	1.074 (0.904-1.276)	.42
ln(GFAP)	1.378 (1.160-1.637)	<.001	1.165 (0.953-1.424)	.14	1.170 (0.951-1.438)	.14	1.167 (0.951-1.433)	.14	1.150 (0.935-1.413)	.19

Abbreviations: Aβ, amyloid beta; GFAP, glial fibrillary acidic protein; ; ln, log transformed; NfL, neurofilament light chain; OR, odds ratio; ptau, phosphorylated tau.

^a^ Results are derived from survey-weighted logistic regression estimates, 95% CIs, and *P* values for associations between natural log-transformed plasma biomarkers and cognitive complaints across 5 nested models using data from SOL-INCA.

^b^ Model 1 is an unadjusted model; model 2 is adjusted for age, sex, education, Hispanic and/or Latino background, and field center; model 3 is additionally adjusted for body mass index, diabetes, hypertension, and dyslipidemia; model 4 is additionally adjusted for chronic kidney disease; model 5 is additionally adjusted for *APOE* genotype.

### Stratified and Sensitivity Analyses

No significant effect modification by age group was found for the associations between plasma biomarkers and ECog-12 scores (eTable 4 in Supplement 1). We observed no consistent evidence of effect modification by sex. The only significant interaction was between ln(GFAP) and ECog-12 memory scores (*F*_1_ = 4.30; *P* = .04) (eTable 4 in Supplement 1).

Cognitive concerns modified associations between ln(NfL) and ECog-12 global (*F*_1_ = 9.91; *P* = .002), executive (*F*_1_ = 10.05; *P* = .001), and memory (*F*_1_ = 4.90; *P* = .03) scores (eTable 4 in Supplement 1). Among those with cognitive concerns, ln(NfL) was associated with higher ECog-12 global (unstandardized β = 0.208; 95% CI, 0.090-0.325; *P* = .001), executive (unstandardized β = 0.231; 95% CI, 0.109-0.354; *P* < .001), and memory (unstandardized β = 0.174; 95% CI, 0.060-0.288; *P* = .003) scores (eTable 5 and eFigure 3 in Supplement 1). No significant associations were found in those without cognitive concerns. Among cognitively unimpaired individuals, higher ln(NfL) was associated with higher ECog-12 global (unstandardized β = 0.105; 95% CI, 0.022-0.189; *P* = .01), executive (unstandardized β = 0.112; 95% CI, 0.037-0.187; *P* = .003), and memory (unstandardized β = 0.099; 95% CI, 0.009-0.188; *P* = .03) scores (eTable 6 in Supplement 1).

### Supplemental Analysis

Findings were consistent when using generalized linear models (log link, gamma family; eTable 7 in Supplement 1). Additional models adjusting for depressive symptoms and anxiety state also yielded similar findings (eTable 8 in Supplement 1).

## Discussion

In this large, multisite study of middle-aged and older Hispanic and/or Latino adults living in the US, higher plasma concentrations of ptau-181, NfL, and GFAP were associated with greater SCD. Specifically, higher ptau-181 was modestly associated with subjective declines in memory. Second, higher plasma NfL, a marker of neuroaxonal damage and/or neurodegeneration, was associated with higher subjective declines in global cognition, executive function, and memory. Third, higher plasma GFAP, a marker of neuroinflammation or glial activation, was associated with greater subjective declines in global cognition and executive function. These associations remained significant after adjusting for sociodemographic, cardiometabolic, kidney-related factors, and *APOE* genotype. Although plasma Aβ_42/40_ was not significantly associated with self-reported cognitive concerns overall, the associations between NfL and subjective declines in global cognition, executive function, and memory were more pronounced among individuals with cognitive concerns. No significant associations were observed between Aβ_42/40_ and ECog-12 outcomes. These findings suggest that assessing cognitive concerns and domain-specific SCD assessments may complement biomarker assessments to inform clinicians on early detection of ADRDs.

In a clinical examination encounter, cognitive concerns can be an early indicator of cognitive or functional decline. While cognitive concerns are associated with an increased likelihood of cognitive decline or conversion to dementia,^23^ brief single-item measures may not capture the full spectrum of subjective changes. The ECog-12, a domain-specific assessment that is one of the most common assessments for SCD, may offer insights into the functional changes associated with underlying neuropathology. In our study, biomarkers specific to neuroaxonal damage and glial activation, NfL and GFAP, were associated with greater SCD assessed via the ECog-12, but not with cognitive concerns alone. Moreover, cognitive concerns modified the association between plasma NfL and subjective reports of global, executive function, and memory decline, with more pronounced associations observed in those reporting cognitive concerns, suggesting that those individuals may have stronger biological underpinnings tied to neuronal damage, making them a more biologically at-risk group. These individuals may benefit from closer monitoring or early interventions. This finding underscores the importance of complementing brief cognitive concerns questions with more detailed assessments.

Our findings align with evidence that SCD is a promising early marker of cognitive decline, although its utility may vary by ethnicity and race. Prior research in this population has shown that SCD is associated with objective cognitive change,^7^ and in a multiracial community sample that includes Caribbean Hispanic individuals, SCD is associated with prodromal dementia.^38^ Prior research form the A4 study^39^ suggest that the association between AD biomarkers (using amyloid-PET) and SCD (using the Cognitive Function Index) is modified by race and ethnicity, which would suggest that the utility of SCD may not be uniform across racial or ethnic groups. Moreover, the A4 study showed that amyloid positivity was associated with greater SCD across racial and ethnic groups, but amyloid-PET was not associated with greater self-report of SCD among Hispanic and/or Latino individuals. Notably, the A4 study examined older adults (aged 65-85 years) and used amyloid-PET, whereas our study includes middle-aged and older adults (aged 50-86 years), and assessed plasma Aβ_42/40_. Aβ_42/40_ was not associated with SCD in our study, which may reflect its low dynamic range and limited sensitivity to detect early amyloid pathology.^40^ In contrast, plasma ptau-181, which is associated with both tau-PET and amyloid-PET,^41^ was modestly associated with subjective memory decline. Neuroimaging studies have shown that early AD is marked by neuronal hyperactivation detectable via fMRI, even before overt clinical symptoms, including among those with SCD, and those with in vivo AD pathology.^42,43,44,45,46,47^ These results underscore the potential utility of plasma ptau-181 as a marker of early tau-related pathology in individuals reporting subjective memory decline.

As ADRD prevention and intervention efforts increasingly focus on early stage disease, understanding SCD in midlife has become a public health priority. In our study, age did not modify associations between plasma biomarkers and SCD, suggesting that SCD during midlife is just as crucial for prevention and treatment strategies at the individual patient and population levels. Among cognitively unimpaired individuals, NfL was associated with subjective global, executive, and memory decline, raising the possibility that early neuroaxonal injury may play a role in perceived cognitive changes that precede overt cognitive impairment. These findings emphasize that NfL as a valuable tool in preclinical ADRDs, and the need to consider diverse populations in biomarker-based research.

### Limitations

There are some limitations to consider in evaluating the results of this study. First, the cross-sectional design limits our ability to infer temporality or causality between plasma biomarker levels and SCD. Second, informant-based reports, which could help validate self-report SCD, were not available at visit 2. Third, plasma biomarkers such as NfL and GFAP are not specific to AD and may be influenced by peripheral processes unrelated to central pathology or the result of other underlying conditions. Fourth, effect sizes are modest in magnitude, suggesting limited clinical relevance at the individual level and the need for longitudinal validation. Fifth, although the ECog-12 and cognitive concerns are used, other SCD scales may yield different associations. Finally, generalizability is limited to Hispanic and/or Latino adults residing in 4 urban US areas, though the study’s complex sampling design enhances representativeness within this population. Despite these limitations, there are significant strengths. This is the largest community-based cohort study of varied Hispanic and/or Latino individuals living in the US, and to the best of our knowledge, the only study that has evaluated the association between plasma biomarkers and SCD (as assessed by the ECog-12).

## Conclusions

In this large, community-based study of middle-aged and older Hispanic and/or Latino adults, higher plasma levels of NfL, GFAP, and ptau-181, but not Aβ_42/40_, were associated with greater SCD. Associations between NfL and SCD were more pronounced among individuals with cognitive concerns, suggesting these concerns may reflect neurodegeneration. These findings highlight the importance of combining biomarker assessments with SCD to improve early identification of ADRD in underrepresented populations.

## Notes

Supplement 1. **eTable 1.** Descriptive Statistics of Plasma Biomarkers and ECog-12 Scores **eTable 2.** Associations Between Standardized Plasma Biomarkers and Subjective Cognitive Decline (ECog-12), SOL-INCA Population **eTable 3.** Associations Between Standardized Plasma Biomarkers and Self-Reported Cognitive Concerns Using Survey Weighted Logistic Regression, SOL-INCA Population **eTable 4.** Tests of Estimated Interactions of the Associations Between Natural Log-Transformed Plasma Biomarkers and Subjective Cognitive Decline (ECog-12) by Age, Sex, and Cognitive Concerns in the Overall SOL-INCA Population **eTable 5.** Associations Between Natural Log-Transformed Plasma Biomarkers and Subjective Cognitive Decline, Stratified by Cognitive Concerns in the Overall SOL-INCA Population **eTable 6.** Associations Between Natural Log-Transformed Plasma Biomarkers and SCD (ECog-12) Among Cognitively Unimpaired Individuals **eTable 7.** Associations Between Natural Log-Transformed Plasma Biomarkers and SCD (ECog-12) Using a Generalized Linear Model (With a Log Link and Gamma Distribution) in the Overall SOL-INCA Population **eTable 8.** Associations Between Natural Log-Transformed Plasma Biomarkers and Subjective Cognitive Decline (ECog-12), SOL-INCA Population **eFigure 1.** Schematic of Inclusion and Exclusion Criteria **eFigure 2.** Linear Associations Between Plasma Biomarkers and Subjective Cognitive Decline (ECog-12) **eFigure 3.** Associations Between Neurofilament Light and Subjective Cognitive Decline (ECog-12) Stratified by Self-Reported Cognitive Concerns

Supplement 2.
Data Sharing Statement
