# Supplementary material for: Alzheimer Disease Biomarkers and Subjective Cognitive Decline Among Hispanic and/or Latino Adults
Source: JAMA Netw Open. 2025 Sep 5;8(9):e2531038. doi: 10.1001/jamanetworkopen.2025.31038 (PMC12413646; doi:10.1001/jamanetworkopen.2025.31038)
Supplement: Supplement 1. — eTable 1. Descriptive Statistics of Plasma Biomarkers and ECog-12 Scores eTable 2. Associations Between Standardized Plasma Biomarkers and Subjective Cognitive Decline (ECog-12), SOL-INCA Population eTable 3. Associations Between Standardized Plasma Biomarkers and Self-Reported Cognitive Concerns Using Survey Weighted Logistic Regression, SOL-INCA Population eTable 4. Tests of Estimated Interactions of the Associations Between Natural Log-Transformed Plasma Biomarkers and Subjective Cognitive Decline (ECog-12) by Age, Sex, and Cognitive Concerns in the Overall SOL-INCA Population eTable 5. Associations Between Natural Log-Transformed Plasma Biomarkers and Subjective Cognitive Decline, Stratified by Cognitive Concerns in the Overall SOL-INCA Population eTable 6. Associations Between Natural Log-Transformed Plasma Biomarkers and SCD (ECog-12) Among Cognitively Unimpaired Individuals eTable 7. Associations Between Natural Log-Transformed Plasma Biomarkers and SCD (ECog-12) Using a Generalized Linear Model (With a Log Link and Gamma Distribution) in the Overall SOL-INCA Population eTable 8. Associations Between Natural Log-Transformed Plasma Biomarkers and Subjective Cognitive Decline (ECog-12), SOL-INCA Population eFigure 1. Schematic of Inclusion and Exclusion Criteria eFigure 2. Linear Associations Between Plasma Biomarkers and Subjective Cognitive Decline (ECog-12) eFigure 3. Associations Between Neurofilament Light and Subjective Cognitive Decline (ECog-12) Stratified by Self-Reported Cognitive Concerns [file jamanetwopen-e2531038-s001.pdf]

Supplemental Online Content

Márquez F, Tarraf W, Gonzalez K, et al. Alzheimer dsease biomarkers and subjective cognitive decline among Hispanic/Latino adults. *JAMA Netw Open*. 2025;8(9):e2531038. doi:10.1001/jamanetworkopen.2025.31038

eTable 1. Descriptive Statistics of Plasma Biomarkers and ECog-12 Scores
eTable 2. Associations Between Standardized Plasma Biomarkers and Subjective Cognitive Decline (ECog-12), SOL-INCA Population
eTable 3. Associations Between Standardized Plasma Biomarkers and Self-Reported Cognitive Concerns Using Survey Weighted Logistic Regression, SOL-INCA Population
eTable 4. Tests of Estimated Interactions of the Associations Between Natural Log-Transformed Plasma Biomarkers and Subjective Cognitive Decline (ECog-12) by Age, Sex, and Cognitive Concerns in the Overall SOL-INCA Population
eTable 5. Associations Between Natural Log-Transformed Plasma Biomarkers and Subjective Cognitive Decline, Stratified by Cognitive Concerns in the Overall SOL-INCA Population
eTable 6. Associations Between Natural Log-Transformed Plasma Biomarkers and SCD (ECog-12) Among Cognitively Unimpaired Individuals
eTable 7. Associations Between Natural Log-Transformed Plasma Biomarkers and SCD (ECog-12) Using a Generalized Linear Model (With a Log Link and Gamma Distribution) in the Overall SOL-INCA Population
eTable 8. Associations Between Natural Log-Transformed Plasma Biomarkers and Subjective Cognitive Decline (ECog-12), SOL-INCA Population
eFigure 1. Schematic of Inclusion and Exclusion Criteria
eFigure 2. Linear Associations Between Plasma Biomarkers and Subjective Cognitive Decline (ECog-12)
eFigure 3. Associations Between Neurofilament Light and Subjective Cognitive Decline (ECog-12) Stratified by Self-Reported Cognitive Concerns

This supplemental material has been provided by the authors to give readers additional information about their work.

© 2025 Márquez F et al. *JAMA Network Open*.

© 2025 Márquez F et al. *JAMA Network Open*.

eTable 1. Descriptive Statistics of Plasma Biomarkers and ECog-12 Scores

	Mean	SD	Min	25th	50th	75th	Max
**ln(A$\beta_{42/40}$)**	-2.69	0.24	-4.15	-2.80	-2.66	-2.54	1.24
**ln(ptau-181)**	0.57	0.48	-1.07	0.26	0.51	0.80	4.26
**ln(NfL)**	2.80	0.57	0.66	2.42	2.75	3.11	6.55
**ln(GFAP)**	4.96	0.49	2.96	4.65	4.95	5.27	7.32
**Aβ42/40**	0.07	0.01	0.02	0.06	0.07	0.08	0.18
**Ptau 181**	2.03	1.70	0.34	1.30	1.66	2.23	70.87
**NfL**	20.07	20.97	1.93	11.27	15.63	22.38	702.75
**GFAP**	160.24	86.12	19.25	104.72	141.36	194.36	1513.36
**ECog-12 Global**	1.54	0.57	1.00	1.08	1.33	1.75	4.00
**ECog-12 Executive**	1.46	0.59	1.00	1.00	1.00	2.00	4.00
**ECog-12 Memory**	1.76	0.68	1.00	1.00	1.00	1.5	4.00

Note 1: SD = standard deviation, Min = minimum, Max = maximum, Aβ = amyloid beta, ptau = phosphorylated tau, NfL = Neurofilament light chain, GFAP = glial fibrillary acidic protein; Note 2: All values are reported in units corresponding to the original assay measurements, except for natural log-transformed (ln) values.

© 2025 Márquez F et al. *JAMA Network Open*.

eTable 2. Associations Between Standardized Plasma Biomarkers and Subjective Cognitive Decline (ECog-12), SOL-INCA Population (unweighted N=5,712).

	**ECog-12 Global**				
	**M1**	**M2**	**M3**	**M4**	**M5**
	**b [95% CI]**	**b [95% CI]**	**b [95% CI]**	**b [95% CI]**	**b [95% CI]**
A$\beta_{42/40}$ (per SD)	-0.029 [-0.065;0.008] (0.120)	-0.016 [-0.052;0.020] (0.376)	-0.011 [-0.047;0.026] (0.570)	-0.008 [-0.045;0.028] (0.649)	-0.005 [-0.041;0.032] (0.806)
ptau-181 (per SD)	0.039 [0.007;0.072] (0.017)	0.026 [-0.005;0.057] (0.101)	0.020 [-0.011;0.051] (0.206)	0.018 [-0.013;0.049] (0.261)	0.015 [-0.016;0.046] (0.353)
NfL (per SD)	0.102 [0.033;0.170] (0.004)	0.079 [0.017;0.141] (0.012)	0.074 [0.014;0.134] (0.016)	0.074 [0.011;0.138] (0.022)	0.074 [0.011;0.136] **(0.022)**
GFAP (per SD)	0.104 [0.061;0.147] (0.000)	0.065 [0.018;0.112] (0.007)	0.072 [0.026;0.117] (0.002)	0.070 [0.024;0.115] (0.003)	0.064 [0.018;0.110] **(0.006)**
	**ECog-12 Executive**				
	**M1**	**M2**	**M3**	**M4**	**M5**
	**b [95% CI]**	**b [95% CI]**	**b [95% CI]**	**b [95% CI]**	**b [95% CI]**
A$\beta_{42/40}$ (per SD)	-0.024 [-0.060;0.013] (0.200)	-0.011 [-0.048;0.025] (0.544)	-0.006 [-0.043;0.032] (0.771)	-0.002 [-0.039;0.036] (0.932)	0.001 [-0.037;0.038] (0.973)
ptau-181 (per SD)	0.045 [0.010;0.080] (0.012)	0.032 [-0.001;0.066] (0.061)	0.026 [-0.007;0.060] (0.127)	0.020 [-0.014;0.054] (0.241)	0.018 [-0.016;0.052] (0.305)
NfL (per SD)	0.106 [0.039;0.172] (0.002)	0.087 [0.025;0.149] (0.006)	0.083 [0.024;0.143] (0.006)	0.080 [0.019;0.141] (0.011)	0.079 [0.018;0.141] **(0.011)**
GFAP (per SD)	0.094 [0.047;0.140] (0.000)	0.068 [0.018;0.118] (0.008)	0.076 [0.027;0.125] (0.002)	0.071 [0.023;0.119] (0.004)	0.068 [0.020;0.117] **(0.006)**
	**ECog-12 Memory**				
	**M1**	**M2**	**M3**	**M4**	**M5**
	**b [95% CI]**	**b [95% CI]**	**b [95% CI]**	**b [95% CI]**	**b [95% CI]**
A$\beta_{42/40}$ (per SD)	-0.021 [-0.058;0.016] (0.275)	-0.016 [-0.052;0.020] (0.378)	-0.013 [-0.050;0.023] (0.473)	-0.013 [-0.050;0.023] (0.471)	-0.010 [-0.047;0.027] (0.601)
ptau-181 (per SD)	0.035 [0.001;0.068] (0.041)	0.025 [-0.008;0.058] (0.143)	0.022 [-0.012;0.055] (0.204)	0.025 [-0.008;0.058] (0.145)	0.022 [-0.011;0.054] (0.194)

© 2025 Márquez F et al. *JAMA Network Open*.

NfL (per SD)	0.089 [0.029;0.148] (0.003)	0.072 [0.016;0.128] (0.011)	0.068 [0.013;0.123] (0.015)	0.073 [0.014;0.131] (0.015)	0.072 [0.015;0.129] **(0.014)**
GFAP (per SD)	0.063 [0.024;0.102] (0.002)	0.030 [-0.013;0.073] (0.172)	0.032 [-0.011;0.075] (0.145)	0.032 [-0.011;0.076] (0.143)	0.026 [-0.017;0.070] (0.234)

Results are derived from survey-weighted linear regression estimates, 95% confidence intervals (CI), and p-values (in parentheses) for associations between standardized plasma biomarkers and ECog-12 scores (global, executive, memory) across five nested models using data from the Study of Latinos-Investigation of Neurocognitive Aging (SOL-INCA; unweighted n =5,712); Note 1: Note 2: ECog-12 = 12-Item form of the Everyday Cognition Scale, Global=Global Cognition, Executive= Executive Function, β = beta, Aβ = amyloid beta, ptau = phosphorylated tau, NfL = Neurofilament light chain, GFAP = glial fibrillary acidic protein. Note 3: M1 is an unadjusted model, M2 is adjusted for adjusted for age, sex, education, Hispanic/Latino background, field center, M3 is additionally adjusted for body mass index, diabetes, hypertension, and dyslipidemia, M4 is additionally adjusted for chronic kidney disease, M5 is additionally adjusted for APOE genotype; Note 4: Bold values denote statistical significance.

© 2025 Márquez F et al. *JAMA Network Open*.

eTable 3. Associations Between Standardized Plasma Biomarkers and Self-Reported Cognitive Concerns Using Survey Weighted Logistic Regression, SOL-INCA Population (unweighted N=5,712).

	**Cognitive Concerns**				
	**M1**	**M2**	**M3**	**M4**	**M5**
	**OR [95% CI]**	**OR [95% CI]**	**OR [95% CI]**	**OR [95% CI]**	**OR [95% CI]**
A$\beta_{42/40}$ (per SD)	0.995 [0.922;1.074]<br>(0.899)	1.015 [0.940;1.097]<br>(0.697)	1.021 [0.945;1.103]<br>(0.603)	1.021 [0.944;1.104]<br>(0.598)	1.029 [0.952;1.113]<br>(0.472)
ptau-181 (per SD)	1.018 [0.937;1.107]<br>(0.667)	1.008 [0.932;1.090]<br>(0.846)	1.001 [0.929;1.080]<br>(0.973)	0.998 [0.928;1.073]<br>(0.953)	0.992 [0.925;1.065]<br>(0.833)
NfL (per SD)	1.056 [0.920;1.212]<br>(0.439)	1.027 [0.919;1.148]<br>(0.638)	1.016 [0.916;1.128]<br>(0.761)	1.015 [0.911;1.130]<br>(0.791)	1.015 [0.911;1.130]<br>(0.789)
GFAP (per SD)	1.166 [1.067;1.275]<br>(0.001)	1.071 [0.971;1.181]<br>(0.170)	1.073 [0.971;1.185]<br>(0.166)	1.072 [0.971;1.183]<br>(0.170)	1.060 [0.960;1.171]<br>(0.251)

Results are derived from survey-weighted logistic regression estimates, 95% confidence intervals (CI), and p-values (in parentheses) for associations between standardized plasma biomarkers and cognitive concerns across five nested models using data from the Study of Latinos-Investigation of Neurocognitive Aging (SOL-INCA unweighted n =5,712); Note 1: All variables are z-scored; Note 2: OR = odds ratio, CI = confidence interval, β = beta, Aβ = amyloid beta, ptau = phosphorylated tau, NfL = Neurofilament light chain, GFAP = glial fibrillary acidic protein. Note 3: M1 is an unadjusted model, M2 is adjusted for adjusted for age, sex, education, Hispanic/Latino background, field center, M3 is additionally adjusted for body mass index, diabetes, hypertension, and dyslipidemia, M4 is additionally adjusted for chronic kidney disease, M5 is additionally adjusted for APOE genotype.

© 2025 Márquez F et al. *JAMA Network Open*.

eTable 4. Tests of Estimated Interactions of the Associations Between Natural Log-Transformed Plasma Biomarkers and Subjective Cognitive Decline (ECog-12) by Age, Sex, and Cognitive Concerns in the Overall SOL-INCA Population

	**Age**			**Sex**			**Cognitive Concerns**		
**ECog-12 Global**									
	**F**	**p-val**	**df**	**F**	**p-val**	**df**	**F**	**p-val**	**df**
**ln(A$\beta_{42/40}$)**	2.44	0.12	1	0.02	0.88	1	0.78	0.38	1
**ln(ptau-181)**	0.09	0.77	1	0.18	0.68	1	0.01	0.93	1
**ln(NfL)**	0.98	0.32	1	0.41	0.52	1	9.91	**<0.01**	1
**ln(GFAP)**	1.24	0.27	1	2.12	0.15	1	1.38	0.24	1
**Executive**									
	**F**	**p-val**	**df**	**F**	**p-val**	**df**	**F**	**p-val**	**df**
**ln(A$\beta_{42/40}$)**	3.37	0.07	1	0.02	0.9	1	0.28	0.59	1
**ln(ptau-181)**	0.24	0.62	1	0.38	0.54	1	0.29	0.59	1
**ln(NfL)**	1.52	0.22	1	0	0.96	1	10.05	**<0.01**	1
**ln(GFAP)**	0.80	0.37	1	1.20	0.27	1	1.87	0.17	1
**Memory**									
	**F**	**p-val**	**df**	**F**	**p-val**	**df**	**F**	**p-val**	**df**
**ln(A$\beta_{42/40}$)**	0.44	0.51	1	0.25	0.62	1	1.50	0.22	1
**ln(ptau-181)**	0.05	0.83	1	0.44	0.51	1	0.32	0.57	1
**ln(NfL)**	0.64	0.42	1	2.71	0.1	1	4.90	**0.03**	1
**ln(GFAP)**	0.38	0.54	1	4.30	**0.04**	1	0.50	0.48	1

Results are derived from survey-weighted linear regression models evaluating interactions between natural log-transformed (ln) plasma biomarkers and ECog-12 scores (global, executive, memory) using data from the Study of Latinos-Investigation of Neurocognitive Aging (SOL-INCA unweighted n =5,712); Note 1: Aβ = amyloid beta, NfL = Neurofilament light chain, ECog-12 = 12-Item form of the Everyday Cognition Scale, Global=Global Cognition, Executive= Executive Function, df=degree of freedom, F=f-test; Note 2: Adjusted models include the following covariates: age, sex, education, Hispanic/Latino background, field center, body mass index, diabetes, hypertension, and dyslipidemia, APOE genotype; Note 3: Bold values denote statistical significance

© 2025 Márquez F et al. *JAMA Network Open*.

eTable 5. Associations Between Natural Log-Transformed Plasma Biomarkers and Subjective Cognitive Decline, Stratified by Cognitive Concerns in the Overall SOL-INCA Population

	**No Concerns**	**Concerns**	**No Concerns**	**Concerns**	**No Concerns**	**Concerns**
	**ECog-12 Global**		**ECog-12 Executive**		**ECog-12 Memory**	
	**Adjusted**	**Adjusted**	**Adjusted**	**Adjusted**	**Adjusted**	**Adjusted**
	**b [95% CI]**	**b [95% CI]**	**b [95% CI]**	**b [95% CI]**	**b [95% CI]**	**b [95% CI]**
**ln(NfL)**	0.051 [-0.014;0.116] (0.121)	0.208 [0.090;0.325] **(0.001)**	0.047 [-0.014;0.108] (0.129)	0.231 [0.109;0.354] **(<0.001)**	0.076 [-0.002;0.153] (0.056)	0.174 [0.060;0.288] **(0.003)**

Results are derived from survey-weighted linear regression estimates, 95% confidence intervals (CI), and p-values (in parentheses) for the associations between natural log-transformed (ln) plasma biomarkers and ECog-12 scores (global, executive, memory) using data from the Study of Latinos-Investigation of Neurocognitive Aging (SOL-INCA unweighted n =5,712) stratified by cognitive concerns; Note 1: ECog-12 = 12-Item form of the Everyday Cognition Scale, Global=Global Cognition, Executive= Executive Function, Aβ = amyloid beta, NfL = Neurofilament light chain, Note 2: M1 is an unadjusted model, M2 is adjusted for adjusted for age, sex, education, Hispanic/Latino background, field center, M3 is additionally adjusted for body mass index, diabetes, hypertension, and dyslipidemia, M4 is additionally adjusted for chronic kidney disease, M5 is additionally adjusted for APOE genotype; Note 3: Bold values denote statistical significance.

© 2025 Márquez F et al. *JAMA Network Open*.

eTable 6. Associations Between Natural Log-Transformed Plasma Biomarkers and SCD (ECog-12) Among Cognitively Unimpaired Individuals

	**ECog-12 Global**	**ECog-12 Executive**	**ECog-12 Memory**
	**M5**	**M5**	**M5**
	**b [95% CI]**	**b [95% CI]**	**b [95% CI]**
ln($A\beta_{42/40}$)	-0.033 [-0.185;0.119] (0.673)	-0.013 [-0.175;0.149] (0.878)	-0.031 [-0.182;0.121] (0.689)
ln(ptau-181)	0.028 [-0.044;0.099] (0.448)	0.041 [-0.033;0.115] (0.279)	0.041 [-0.036;0.118] (0.297)
ln(NfL)	0.105 [0.022;0.189] **(0.014)**	0.112 [0.037;0.187] **(0.003)**	0.099 [0.009;0.188] **(0.031)**
ln(GFAP)	0.042 [-0.041;0.126] (0.317)	0.054 [-0.027;0.136] (0.191)	-0.009 [-0.100;0.081] (0.840)

Results are derived from survey-weighted linear regression estimates, 95% confidence intervals (CI), and p-values (in parentheses) for the associations between natural log-transformed (ln) plasma biomarkers and ECog-12 scores (global, executive, memory)using data from the Study of Latinos-Investigation of Neurocognitive Aging (SOL-INCA unweighted n =5,712); Note 1: ECog-12 = 12-Item form of the Everyday Cognition Scale, Global=Global Cognition, Executive= Executive Function, β = beta, Aβ = amyloid beta, ptau = phosphorylated tau, NfL = Neurofilament light chain, GFAP = glial fibrillary acidic protein; Note 2: models (M5) are fully adjusted and include the following covariates: age, sex, education, Hispanic/Latino background, field center, body mass index, diabetes, hypertension, and dyslipidemia, APOE genotype. Note 3: Bold values denote statistical significance.

© 2025 Márquez F et al. *JAMA Network Open*.

eTable 7. Associations Between Natural Log-Transformed Plasma Biomarkers and SCD (ECog-12) Using a Generalized Linear Model (With a Log Link and Gamma Distribution) in the Overall SOL-INCA Population

	ECog-12 Global				
	M1	M2	M3	M4	M5
	Exp(b) [95% CI]	Exp(b) [95% CI]	Exp(b) [95% CI]	Exp(b) [95% CI]	Exp(b) [95% CI]
ln(A$\beta_{42/40}$)	0.950 [0.900;1.002] (0.058)	0.968 [0.919;1.019] (0.214)	0.974 [0.925;1.026] (0.317)	0.976 [0.927;1.028] (0.354)	0.980 [0.931;1.032] (0.439)
ln(ptau-181)	1.049 [1.020;1.078] (0.001)	1.034 [1.004;1.064] (0.024)	1.029 [1.000;1.059] (0.047)	1.028 [0.999;1.059] (0.062)	1.025 [0.996;1.056] (0.091)
ln(NfL)	1.088 [1.058;1.118] (<0.001)	1.064 [1.033;1.095] (<0.001)	1.059 [1.028;1.091] (<0.001)	1.061 [1.028;1.096] (<0.001)	1.061 [1.028;1.095] **(<0.001)**
ln(GFAP)	1.075 [1.045;1.107] (<0.001)	1.040 [1.007;1.073] (0.016)	1.045 [1.013;1.078] (0.006)	1.043 [1.011;1.077] (0.008)	1.040 [1.007;1.073] **(0.017)**
	**ECog-12 Executive**				
	M1	M2	M3	M4	M5
	Exp(b) [95% CI]	Exp(b) [95% CI]	Exp(b) [95% CI]	Exp(b) [95% CI]	Exp(b) [95% CI]
ln(A$\beta_{42/40}$)	0.948 [0.890;1.010] (0.097)	0.967 [0.909;1.028] (0.284)	0.975 [0.917;1.037] (0.419)	0.979 [0.920;1.042] (0.502)	0.981 [0.923;1.044] (0.552)
ln(ptau-181)	1.057 [1.024;1.092] (0.001)	1.043 [1.009;1.077] (0.013)	1.038 [1.005;1.072] (0.026)	1.033 [0.999;1.069] (0.059)	1.031 [0.998;1.067] (0.070)
ln(NfL)	1.095 [1.062;1.129] (<0.001)	1.077 [1.043;1.112] (<0.001)	1.073 [1.040;1.108] (<0.001)	1.072 [1.035;1.110] (<0.001)	1.072 [1.036;1.110] **(<0.001)**
ln(GFAP)	1.074 [1.039;1.110] (<0.001)	1.048 [1.012;1.086] (0.008)	1.055 [1.020;1.092] (0.002)	1.051 [1.015;1.088] (0.005)	1.049 [1.013;1.086] **(0.007)**
	**ECog-12 Memory**				
	M1	M2	M3	M4	M5
	Exp(b) [95% CI]	Exp(b) [95% CI]	Exp(b) [95% CI]	Exp(b) [95% CI]	Exp(b) [95% CI]
ln(A$\beta_{42/40}$)	0.954 [0.903;1.007] (0.087)	0.962 [0.912;1.014] (0.152)	0.965 [0.916;1.017] (0.181)	0.965 [0.916;1.017] (0.179)	0.969 [0.920;1.021] (0.236)
ln(ptau-181)	1.046 [1.017;1.076] (0.002)	1.037 [1.006;1.070] (0.020)	1.035 [1.004;1.068] (0.029)	1.039 [1.007;1.073] (0.016)	1.036 [1.004;1.069] **(0.027)**
ln(NfL)	1.075 [1.046;1.104] (<0.001)	1.058 [1.026;1.091] (<0.001)	1.054 [1.023;1.087] (0.001)	1.061 [1.027;1.096] (<0.001)	1.060 [1.026;1.095] **(<0.001)**
ln(GFAP)	1.048 [1.017;1.081] (0.002)	1.018 [0.984;1.052] (0.304)	1.019 [0.986;1.054] (0.269)	1.019 [0.986;1.054] (0.263)	1.015 [0.981;1.050] (0.398)

© 2025 Márquez F et al. *JAMA Network Open*.

Results are derived from survey-weighted generalized linear model estimates (log link and gamma family), 95% confidence intervals (CI), and p-values (in parentheses) for the associations between natural log-transformed plasma biomarkers and ECog-12 scores (global, executive, memory) using data from the Study of Latinos-Investigation of Neurocognitive Aging (SOL-INCA unweighted n =5,712); Note 1: ECog-12 = 12-Item form of the Everyday Cognition Scale, Global=Global Cognition, Executive= Executive Function, Aβ = amyloid beta, NfL = Neurofilament light chain, Note 2: M1 is an unadjusted model, M2 is adjusted for adjusted for age, sex, education, Hispanic/Latino background, field center, M3 is additionally adjusted for body mass index, diabetes, hypertension, and dyslipidemia, M4 is additionally adjusted for chronic kidney disease, M5 is additionally adjusted for APOE genotype; Note 3: Bold values denote statistical significance. Note 4: The beta and 95% confidence intervals are exponentiated.

© 2025 Márquez F et al. *JAMA Network Open*.

eTable 8. Associations Between Natural Log-Transformed Plasma Biomarkers and Subjective Cognitive Decline (ECog-12), SOL-INCA Population (unweighted N=5,712).

	**ECog-12 Global**	**ECog-12 Memory**	**ECog-12 Executive**
	**M6**	**M6**	**M6**
	**b [95% CI]**	**b [95% CI]**	**b [95% CI]**
ln(A$\beta_{42/40}$)	-0.091 [-0.240;0.059] (0.233)	-0.082 [-0.247;0.082] (0.327)	-0.107 [-0.257;0.044] (0.164)
ln(ptau-181)	0.065 [-0.011;0.141] (0.094)	0.075 [-0.007;0.158] (0.074)	0.090 [0.010;0.169] **(0.027)**
ln(NfL)	0.154 [0.068;0.239] **(<0.001)**	0.169 [0.080;0.259] **(<0.001)**	0.143 [0.060;0.225] **(<0.001)**
ln(GFAP)	0.106 [0.022;0.190] **(0.013)**	0.117 [0.031;0.204] **(0.008)**	0.042 [-0.042;0.127] (0.327)

Results are derived from survey-weighted linear regression estimates, 95% confidence intervals (CI), and p-values (in parentheses) for associations between natural log-transformed plasma biomarkers and ECog-12 domain scores (global, executive, memory) using data from the Study of Latinos-Investigation of Neurocognitive Aging (SOL-INCA; unweighted n =5,712); Note 1: Note 2: ECog-12 = 12-Item form of the Everyday Cognition Scale, Global=Global Cognition, Executive= Executive Function, β = beta, Aβ = amyloid beta, ptau = phosphorylated tau, NfL = Neurofilament light chain, GFAP = glial fibrillary acidic protein. Note 3: M6 is fully adjusted including age, sex, education, Hispanic/Latino background, field center, body mass index, diabetes, hypertension, and dyslipidemia, for chronic kidney disease, APOE genotype, depression and anxiety; Note 4: Bold values denote statistical significance.

© 2025 Márquez F et al. *JAMA Network Open*.

eFigure 1. Schematic of Inclusion and Exclusion Criteria

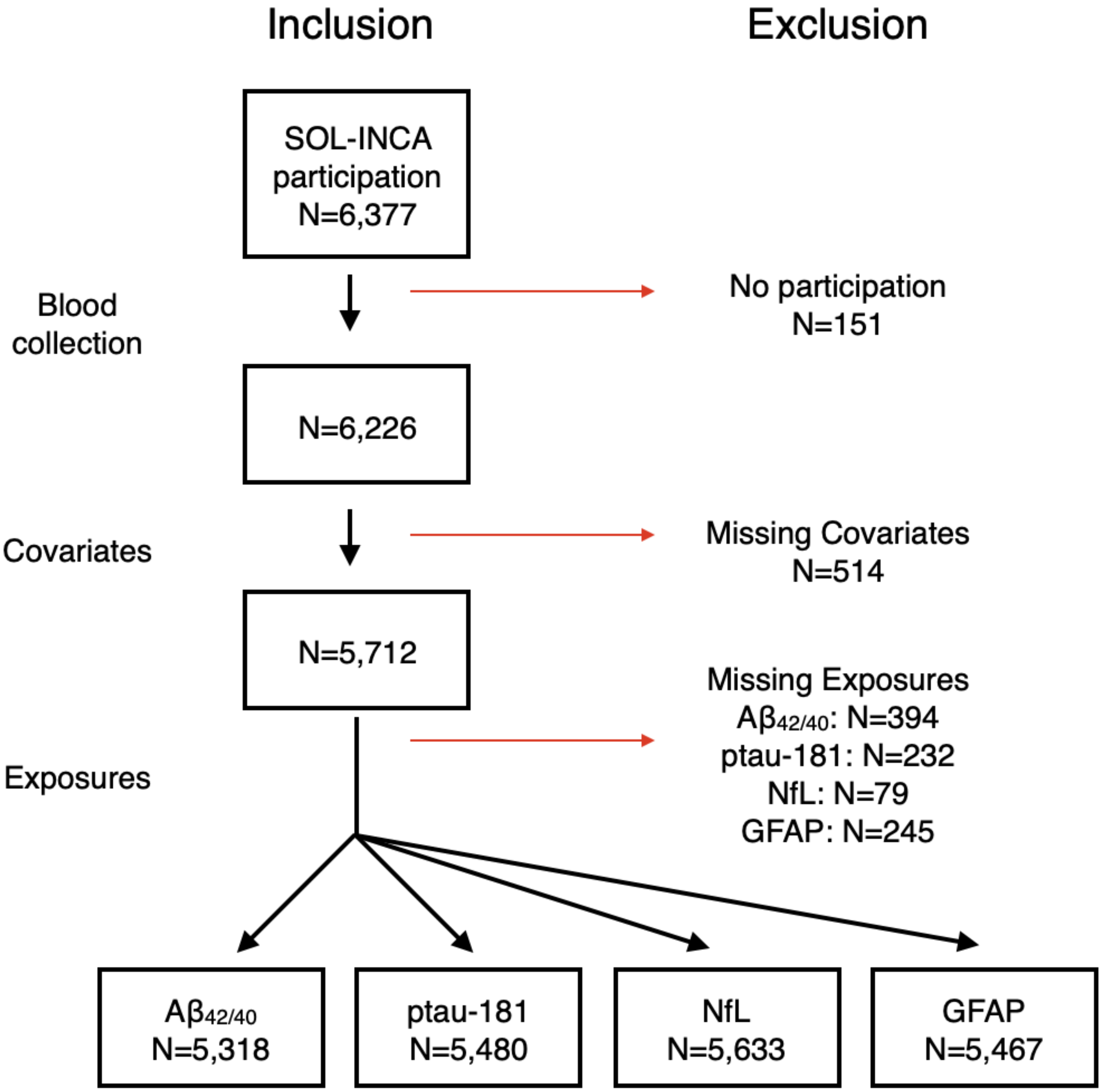

Note 1: Aβ = amyloid beta, ptau = phosphorylated tau, NfL = Neurofilament light chain, GFAP = glial fibrillary acidic protein

© 2025 Márquez F et al. *JAMA Network Open*.

eFigure 2. Linear Associations Between Plasma Biomarkers and Subjective Cognitive Decline (ECog-12)

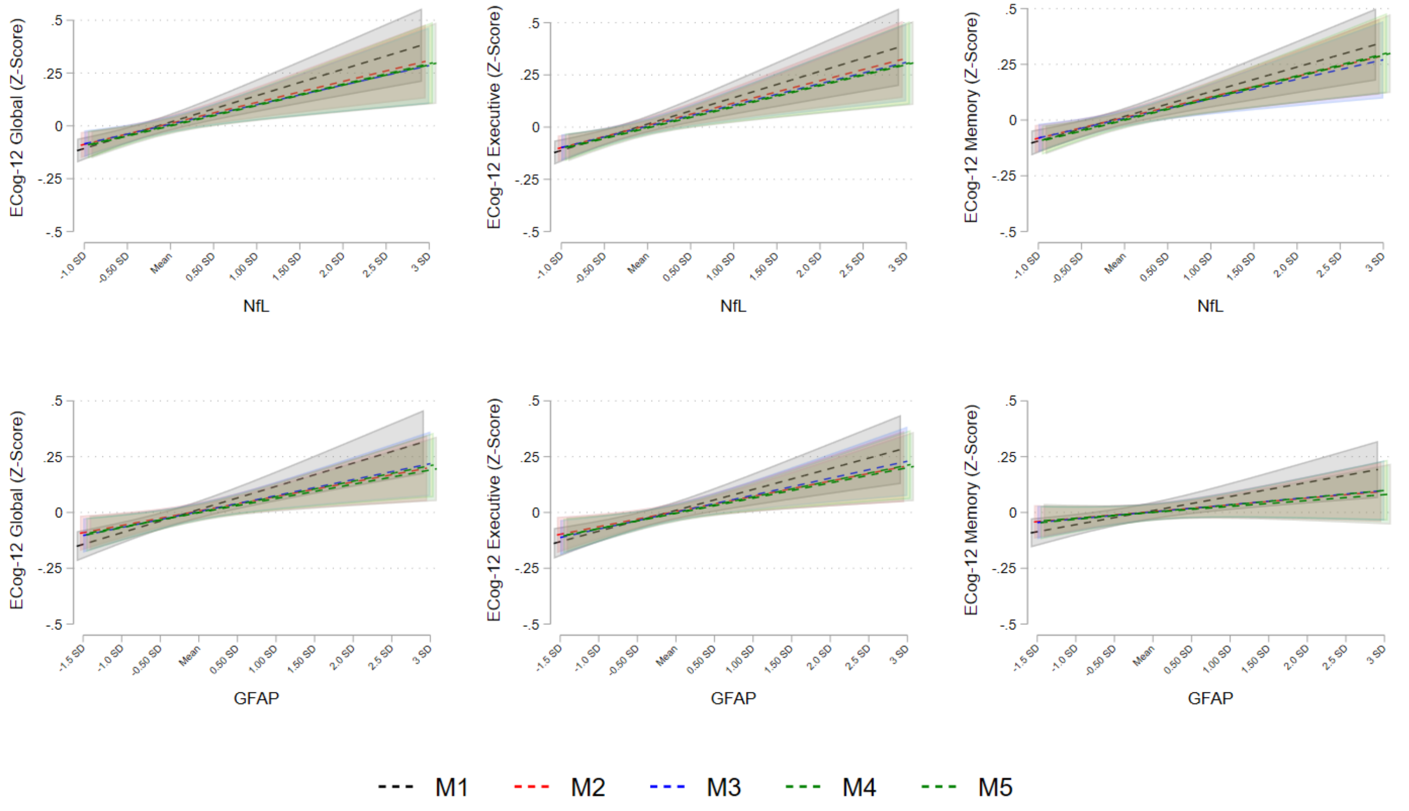

Results are derived from survey-weighted linear regression estimates and 95% confidence intervals (CI) for associations between standardized plasma biomarkers and ECog-12 scores (global, executive, memory) across five nested models using data from the Study of Latinos-Investigation of Neurocognitive Aging (SOL-INCA; unweighted n =5,712); Note 1: Variables on the x-axis are mean centered to facilitate interpretation, and variables on the y-axis are z-scored; Note 2: ECog-12 = 12-Item form of the Everyday Cognition Scale, Global=Global Cognition, Executive= Executive Function, NfL = Neurofilament light chain, GFAP = glial fibrillary acidic protein. Note 3: M1 is an unadjusted model, M2 is adjusted for adjusted for age, sex, education, Hispanic/Latino background, field center, M3 is additionally adjusted for body mass index, diabetes, hypertension, and dyslipidemia, M4 is additionally adjusted for chronic kidney disease, M5 is additionally adjusted for APOE genotype.

© 2025 Márquez F et al. *JAMA Network Open*.

eFigure 3. Associations Between Neurofilament Light and Subjective Cognitive Decline (ECog-12) Stratified by Self-Reported Cognitive Concerns

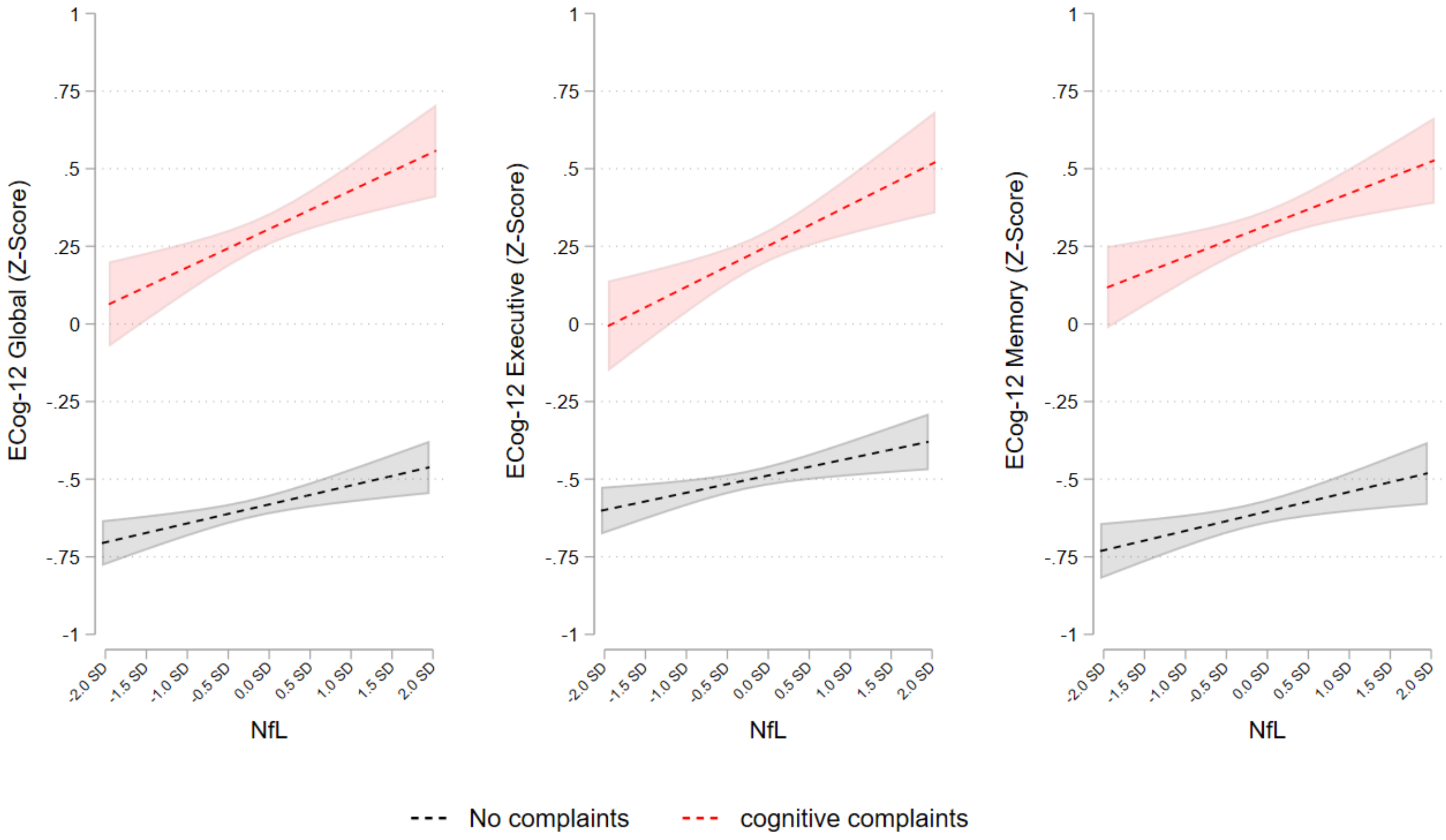

Results are derived from linear regression models using data from the Study of Latinos-Investigation of Neurocognitive Aging (SOL-INCA unweighted n = 5,712); Note 1: Variables on the x-axis are mean centered to facilitate interpretation, and variables on the y-axis are z-scored; Note 2: ECog-12 = 12-Item form of the Everyday Cognition Scale, Global=Global Cognition, Executive= Executive Function, NfL = Neurofilament light chain, Note 3: M1 is a unadjusted model, M2 is adjusted for adjusted for age, sex, education, Hispanic/Latino background, field center, M3 is additionally adjusted for body mass index, diabetes, hypertension, and dyslipidemia, M4 is additionally adjusted for chronic kidney disease, M5 is additionally adjusted for APOE genoty

© 2025 Márquez F et al. *JAMA Network Open*.
