# Supplementary material for: Alzheimer Disease Biomarkers and Subjective Cognitive Decline Among Hispanic and/or Latino Adults
Source: JAMA Netw Open. 2025 Sep 5;8(9):e2531038. doi: 10.1001/jamanetworkopen.2025.31038 (PMC12413646; doi:10.1001/jamanetworkopen.2025.31038)
Supplement: Supplement 2. — Data Sharing Statement [file jamanetwopen-e2531038-s002.pdf]

Data Sharing Statement

Márquez. Alzheimer Disease Biomarkers and Subjective Cognitive Decline Among Hispanic/Latino Adults. *JAMA Netw Open*. Published September 05, 2025. doi:10.1001/jamanetworkopen.2025.31038

Data
Data available: Yes
Data types: Deidentified participant data, Data dictionary
How to access data: https://sites.cscc.unc.edu/hchs/manuals-forms
When available: With publication

Supporting Documents
Document types: None

Additional Information
Who can access the data: Researchers whose proposed use of the data has been approved.
Types of analyses: For specified purpose.
Mechanisms of data availability: With investigator support and after approval of a proposal.
